# *Veronica persica* Ethanol Extract Ameliorates Dinitrochlorobenzene-Induced Atopic Dermatitis-like Skin Inflammation in Mice, Likely by Inducing Nrf2/HO-1 Signaling

**DOI:** 10.3390/antiox12061267

**Published:** 2023-06-13

**Authors:** Ki-Shuk Shim, Musun Park, Won-Kyung Yang, Hanbyeol Lee, Seung-Hyung Kim, Byung-Kil Choo, Sungwook Chae, Ho-Kyoung Kim, Taesoo Kim, Ki-Mo Kim

**Affiliations:** 1 KM Convergence Research Division, Korea Institute of Oriental Medicine, Yuseong-daero 1672, Yuseong-gu, Daejeon 34054, Republic of Korea; angeloshim@kiom.re.kr (K.-S.S.); kendall@kiom.re.kr (S.C.); hkkim@kiom.re.kr (H.-K.K.); 2 KM Data Division, Korea Institute of Oriental Medicine, Yuseong-daero 1672, Yuseong-gu, Daejeon 34054, Republic of Korea; bmusun@kiom.re.kr; 3 Institute of Traditional Medicine and Bioscience, Daejeon University, Daejeon 34520, Republic of Korea; ywks1220@dju.kr (W.-K.Y.); qufzhd0705@naver.com (H.L.); sksh518@dju.kr (S.-H.K.); 4 Department of Crop Science & Biotechnology, Jeonbuk National University, Jeonju 54896, Republic of Korea; bkchoo@jbnu.ac.kr; 5 Korean Convergence Medicine Major KIOM, University of Science & Technology (UST), Daejeon 34054, Republic of Korea

**Keywords:** atopic dermatitis, *Veronica persica*, nuclear factor erythroid 2-related factor 2, heme oxygenase-1

## Abstract

Atopic dermatitis (AD) is chronic allergic contact dermatitis with immune dysregulation. *Veronica persica* has pharmacological activity that prevents asthmatic inflammation by ameliorating inflammatory cell activation. However, the potential effects of the ethanol extract of *V. persica* (EEVP) on AD remain elusive. This study evaluated the activity and underlying molecular pathway of EEVP in two AD models: dinitrochlorobenzene (DNCB)-induced mice and interferon (IFN)-γ/tumor necrosis factor (TNF)-α-stimulated human HaCaT keratinocytes. EEVP attenuated the DNCB-induced increase in serum immunoglobulin E and histamine levels, mast cell counts in toluidine-blue-stained dorsal skin, inflammatory cytokine (IFN-γ, interleukin [IL]-4, IL-5, and IL-13) levels in cultured splenocytes, and the mRNA expression of *IL6*, *IL13*, *IL31 receptor*, *CCR-3*, and *TNFα* in dorsal tissue. Additionally, EEVP inhibited the IFN-γ/TNF-α-induced mRNA expression of *IL6*, *IL13*, and *CXCL10* in HaCaT cells. Furthermore, EEVP restored the IFN-γ/TNF-α-induced downregulation of heme oxygenase (HO)-1 in HaCaT cells by inducing nuclear factor erythroid 2-related factor 2 (Nrf2) expression. A molecular docking analysis demonstrated that EEVP components have a strong affinity to the Kelch-like ECH-associated protein 1 Kelch domain. In summary, EEVP inhibits inflammatory AD by attenuating immune cell activation and inducing the Nrf2/HO-1 signaling pathway in skin keratinocytes.

## 1. Introduction

Atopic dermatitis (AD) is a chronic inflammatory skin disease characterized by several symptoms including pruritic and relapsing eczematous skin lesions with a thickened epidermis and skin barrier defects [1,2]. Skin barrier dysfunction and/or immune dysregulation, which are stimulated by interactions between genetic factors and environmental allergens, have been suggested as the major causes of AD pathogenesis [3,4]. An abnormal cutaneous barrier facilitates environmental exposure and triggers the resident skin immune cells to secrete chemokines and cytokines that activate local or systemic immune responses. Subsequently, the Th1/Th2 immune system balance is perturbed, and an inflammatory cascade associated with AD develops [5]. Typically, glucocorticosteroids, anti-histamines, or immunosuppressive calcineurin inhibitors are used as first-line therapeutics for AD [6,7]. However, the side effects of long-term use of these agents can cause skin atrophy and vulnerability to infection [8]. The biologic antibody, dupilumab, and Janus kinase inhibitor, upadacitinib, which show good efficacy and safety profiles, are emerging treatment options for moderate-to-severe AD [9,10,11]. Researchers are also actively exploring novel therapeutic options based on natural products with minimal adverse reactions for inflammatory skin diseases, such as AD. Several herbal or natural products are reported to suppress either AD or allergic asthma by regulating immune cell imbalance, suggesting that they are alternative therapeutic options [12,13].

The detoxification of exogenous factors, such as irritants or allergens, increases reactive oxygen species (ROS) levels in keratinocytes and results in the attraction and stimulation of eosinophils and macrophages, leading to chronic AD inflammation [14]. The transcription factor nuclear factor erythroid 2-related factor 2 (Nrf2) is a crucial regulator of anti-oxidant proteins such as heme oxygenase 1 (HO-1) [15]. The Nrf2/HO-1 signaling pathway, which activates antioxidant gene expression, protects against oxidation and inflammatory reactions by inhibiting ROS generation [14]. Interleukin (IL)-4, IL-10, and IL-13 upregulate HO-1 expression, and HO-1 reduces pro-inflammatory cytokine production [16,17]. Additionally, HO-1 is highly expressed in resident macrophages and dendritic cells to attenuate local inflammatory responses in AD-like skin lesions of mice [18]. Therefore, the Nrf2/HO-1 signaling pathway may be important for the amelioration of dermatitis in patients with AD.

*Veronica persica*, a plant naturalized mainly from Europe and South Asia, belongs to the Plantaginaceae (formerly Scrophulariaceae) family. The aerial parts of *V. persica* have been used in traditional Chinese medicine to treat genital and kidney diseases. Analytical and pharmacological studies have reported that the iridoid and phenolic components of *V. persica* have ROS-scavenging or anti-inflammatory activities in vitro [19,20,21]. Recently, *V. persica* has been reported to prevent house dust mite-induced asthmatic inflammation by ameliorating inflammatory cell infiltration and activation [22]. AD is often followed by allergic asthma or rhinitis, which are members of the atopic triad of immune dysregulated diseases [23]. The pathophysiological processes of these diseases are similar and include an imbalance in immune cells and an increase in inflammatory mediators and allergic triggers. Therefore, we hypothesized that *V. persica*, which has shown pharmaceutical efficacy in treating asthmatic airway inflammation, may also exhibit therapeutic potential in suppressing AD.

NC/Nga mice are an inbred animal model that can spontaneously develop human AD-like skin lesions [24]. Periodic dinitrochlorobenzene (DNCB) application to the skin of NC/Nga mice induces AD-like skin lesions that exhibit increased serum immunoglobulin (Ig)E levels and eosinophil, mast cell, and Th2 cell populations with chronic skin changes [25]. In addition, tumor necrosis factor (TNF)-α/interferon (IFN)-γ stimulation of HaCaT keratinocytes has been suggested as an in vitro model for cellular and molecular studies on AD [26]. In this study, we explored the potential inhibitory effects of an ethanol extract of *V. persica* (EEVP) on DNCB-induced AD in an NC/Nga murine model. To elucidate the underlying molecular pathway of EEVP activity on AD, we examined the effect of EEVP on the Nrf2/HO-1 signaling pathway in HaCaT human keratinocytes.

## 2. Materials and Methods

### 2.1. Plant Materials

*V. persica* (family Plantaginaceae) was provided by Prof. Byung Kil Choo (Chonbuk National University, Jeonju, Republic of Korea) and was subjected to visual and organoleptic evaluation of the plant material. *V. persica* (10 g) (voucher number: KIOM 52) was extracted by low-temperature boiling in 70% ethanol (100 mL) for 2 h, repeated three times. The resulting crude extract was then concentrated in a vacuum freeze dryer and stored at −20 °C. EEVP was characterized using ultraperformance liquid chromatography/quadrupole-orbitrap mass spectrometry as previously reported [22].

### 2.2. Animal Experiments

The animal experiment procedures were conducted based on the rules issued by the Institutional Animal Care and Use Committee of Daejon University (approval number: DJUARB2021-0033) and in compliance with the Guide for the Care and Use of Laboratory Animals of the National Institutes of Health (2013). The animals were housed, and the experiments were conducted, in a standard laboratory animal facility at 23 ± 2 °C and humidity of 55 ± 10%, with a 12 h light–dark cycle and ad libitum feeding, following the committee guidelines. A DNCB-induced AD mouse model was established in accordance with previous studies [25,27,28,29]. The health status of the mice was monitored throughout the experiment. The mice (6-week-old NC/Nga male mice; Central Lab Animal Inc., Seoul, Republic of Korea) were randomly allocated to four groups (*n* = 6): (1) an NC/Nga normal control group administered with phosphate-buffered saline (PBS), (2) a DNCB-induced AD control group administered with PBS, (3) a DNCB-induced AD group administered with 3 mg/kg dexamethasone (positive control) [28], and (4) a DNCB-induced AD group administered with 200 mg/kg EEVP. The initial body weight and health status did not differ significantly among the groups. The administered dosages of EEVP and dexamethasone were determined based on their effective dosages in previous studies [22,28]. AD was induced by carrying out a dermal application of 200 μL of 1% DNCB solution (acetone:olive oil = 3:1) to the dorsal area of mice after shaving. Three days later, allergic challenge was further induced by painting with 0.5% and 0.2% DNCB solutions three times per week, respectively. The normal control mice orally received the vehicle only. EEVP (200 mg/kg) and dexamethasone (3 mg/kg) were dissolved in water and orally administered daily for 2 weeks with DNCB application. The clinical index of severity of dermatitis was blindly quantified using a five-point grading system [25]. The head and neck, upper extremities, trunk, and lower extremities were assessed for five pathological signs: erythema/hemorrhage, edema/hematoma, excoriation/erosion, itching/dryness, and lichenification (skin thickening). A proportional score ranging from 0 to 6 was designed to indicate the percentage of areas changed in each of the four body regions during the analysis: none (0), 1–9% (1), 10–29% (2), 30–49% (3), 50–69% (4), 70–89% (5), and 90–100% (6). The total clinical index of dermatitis severity was the sum of the graded individual scores.

### 2.3. Analysis of Serum Ig E, Histamine, and Immune Cytokines

Blood samples were obtained from the anesthetized mice via cardiac puncture. After being maintained at room temperature (25 °C) for 30 min, the serum was centrifuged at 3000× *g* for 10 min at 4 °C and collected. The levels of IgE and histamine in the serum and those of IL-4, -5, and -13 and IFN-γ in splenocyte culture supernatants, were measured using mouse ELISA kits specific to each target (R&D Systems, Minneapolis, MN, USA).

### 2.4. Histological Examination of the AD-like Dermis

The collected dorsal skin tissues were incubated with 10% (*v*/*v*) formalin solution (Sigma-Aldrich, Burlington, MA, USA) for fixation. Then, the fixed tissues were embedded in paraffin and sliced into 4 μm thick sections using a Cryostat Microtome (Leica, Wetzlar, Germany). To determine the degree of pathological changes in the dorsal skin lesions, hematoxylin and eosin (H&E) staining was performed. Toluidine blue staining was performed to determine inflammatory mast cell infiltration. At least three different areas of each tissue section were photographed and evaluated under light microscopy (Nikon, Tokyo, Japan). A histopathological analysis was performed for AD-like changes in dorsal skin lesions. Dorsal skin sections were subjected to blind quantification based on dermatitis scores of 0 (none), 1 (mild), 2 (moderate), or 3 (severe) for clinical features using a previously published protocol with modification [30]. The inflammatory cells in the dorsal skin sections were enumerated. Three different areas of each tissue section were examined using light microscopy (magnification: ×100).

### 2.5. Flow Cytometry Analysis

Peripheral blood mononuclear cells (PBMCs) were collected from the heparinized blood using Percoll discontinuous density-gradient centrifugation at 400× *g* for 20 min. Primary cells were isolated from axillary lymph nodes (ALNs) and dorsal skin using collagenase digestion (1 mg/mL) (Sigma-Aldrich, Burlington, MA, USA) for 40 min incubation. The cell suspension was filtered using a 70 μm pore size nylon cell strainer (BD Falcon, Bedford, MA, USA) and then centrifuged at 450× *g* for 20 min. The cell pellet was washed twice with PBS. PBMCs and primary cells were stained with specific fluorescent-conjugated antibodies (anti-CD3, anti-CD4, anti-CD8, anti-CD19, anti-CD23, anti-B220, anti-CD11b, and anti-chemokine–chemokine receptor 3) (BD Biosciences, San Jose, CA, USA) for 10 min on ice in a fluorescence-activated cell sorting buffer (PBS with 3% fetal bovine serum [FBS] and 0.1% sodium azide). After washing with the buffer three times, the stained cells were analyzed using a BD FACSCaliburTM two-color flow cytometer interfaced with the CellQuest software (643274, BD Biosciences). The absolute cell number was determined by dividing the number of cells of interest by the total cell number; the result is expressed as a percentage.

### 2.6. Splenocyte Isolation and Cytokine Measurement

The spleens of the mice were mashed with a medical spatula and filtered into a culture dish using a 70 μm cell strainer. After removing red blood cells (RBCs) via incubation with an RBC lysis buffer for 5 min, the cells were collected using centrifugation. The isolated splenocytes (1 × 10^5^ cells/well; 96-well) were incubated in the presence of samples with RPMI-1640 medium (Thermo Fisher Scientific, Waltham, MA, USA) containing 10% FBS in an anti-CD3 antibody-bound plate (0.5 μg/mL) (R&D Systems) for 48 h. The culture supernatants were examined to determine IL-4, -5, and -13 and IFN-γ levels, using a mouse ELISA kit (R&D Systems).

### 2.7. Quantitative Real-Time Reverse Transcription Polymerase Chain Reaction

The total RNA was isolated from the skin tissues using RNAzol B based on phenol–chloroform phase separation (RNAzol B: Tel-Test Co. Inc., Friendswood, TX, USA). The total RNA was isolated from the HaCaT cells using an RNeasy Mini Kit (Qiagen, Hilden, Germany). The total RNA (5 μg) was reverse-transcribed into cDNA using a first-strand cDNA synthesis kit (Amersham Pharmacia, Piscataway, NJ, USA). The mRNA was amplified using a TaqMan Universal Master Mix II (Applied Biosystems, Foster City, CA, USA) on an ABI 7500 RT-PCR instrument (Applied Biosystems). The qRT-PCR conditions consisted of an incubation step (2 min at 50 °C and 10 min at 94 °C) and an amplification step (40 cycles of 1 min each at 94 °C and 1 min at 60 °C). The relative mRNA expression of the target gene was calculated using the ΔΔCt method. The PCR primer sequences (IL-13, IL-31 receptor, IL-6, chemokine–chemokine receptor 3, TNF-α, and glyceralde-hyde-3-phosphate dehydrogenase) were used in the same way as in a previous study [25].

### 2.8. Cell Culture and Cell Viability Assay

HaCaT human keratinocyte cells were purchased from American Type Culture Collection (Manassas, VA, USA) and cultured in Dulbecco’s Modified Eagle’s Medium containing heat-inactivated 10% FBS and 1% penicillin/streptomycin (Thermo Fisher Scientific). For RNA isolation, the HaCaT cells (0.5 × 10^6^ cells/well, 6-well plate) were incubated with EEVP (400 μg/mL) or dexamethasone (20 μM) for 24 h and then incubated with or without TNF-α/IFN-γ (10 ng/mL) (Thermo Fisher Scientific). Cell cytotoxicity was determined by quantifying the relative formation of formazan in proportion to the live-cell number using a Cell Counting Kit-8 (CCK-8; Dojindo, Kumamoto, Japan). The cells (5 × 10^4^ cells/well) were seeded in 96-well plates, incubated overnight, and treated with several concentrations of EEVP (25–400 μg/mL) for 24 h. The cells were then incubated with the CCK-8 reagent for 1 h. The absorbance of the medium was measured using a microplate reader (450 nm; Molecular Devices, San Jose, CA, USA).

### 2.9. Western Blot Analysis

The HaCaT cells (0.5 × 10^6^ cells/well; 6-well plate) were pre-incubated with EEVP (400 μg/mL) or dexamethasone (20 μM) for 30 min and then incubated with 10 ng/mL of IFN-γ and TNF-α for 2 h. After being washed with PBS, the HaCaT cells were fractionated into cytosol and nuclear fractions using NE-PER Nuclear and Cytoplasmic Extraction Reagents (Thermo Fisher Scientific) containing protease and a phosphatase inhibitor (Thermo Fisher Scientific). The protein concentration was assessed by measuring the colorimetric change of bicinchoninic acid solution in comparison with bovine serum albumin as the standard (Thermo Fisher Scientific). After heating with 2 × sample buffer, the total amount of protein (10–20 μg) in each fractionation was separated using 10% SDS-PAGE gel electrophoresis and transferred onto PVDF membranes using a semidry transfer instrument (2.5 A, 25 V, 7 min) (Bio-Rad, Hercules, CA, USA). The membranes were incubated with EzBlock Chemi (ATTO, Osaka, Japan) for 1 h blocking. Nrf2 (12721), HO-1 (70081), histone H-3 (4499), and β-actin (4967) were obtained from Cell Signaling Technology (Danvers, MA, USA). The membranes were each immersed in a primary antibody solution (1:1000 dilution) for 2 h and then a horseradish peroxidase conjugated rabbit secondary antibody solution (1:5000 dilution) for 1 h. After washing three times with TBST in between antibody incubations, the membranes were reacted with SuperSignal West Femto Chemiluminescent Substrate (Bio-Rad) to detect chemiluminescent signals at the expected molecular weights under a chemiluminescence imaging system (Vilber Lourmat, Marne-la-Vallee, France). The intensities of the bands were analyzed using ImageJ software 1.53 t (NIH, Bethesda, MD, USA).

### 2.10. Docking Analysis of The Kelch-Like ECH-Associated Protein (KEAP)-1 Kelch Domain Pocket and EEVP Components

The chemical structures of catalposide, verproside, picroside, aucubin, loganic acid, and ginnalin A were retrieved from the PubChem database (https://pubchem.ncbi.nlm.nih.gov, accessed on 9 May 2023) [31]. The structure of the human-derived Kelch-like ECH-associated protein (Keap)-1 was collected from the AlphaFold platform (https://alphafold.ebi.ac.uk/) [32]. A molecular docking analysis between the structure of the KEAT1 Kelch domain pocket and six molecules was performed using OpenBabel (v3.1.1) [33], AutoDock Vina API (Python library. V1.1.2) [34], Python (v3.8.12), and Discovery Studio 2021. First, the chemical and protein structures were transformed into the PDBQT format using the OpenBabel software. The docking prediction was performed using Python and AutoDock Vina API, and the analysis parameter exhaustiveness was set to a maximum value of 100. Visualization was performed using Discovery Studio 2021.

### 2.11. Statistical Analysis

Data from the animal experiments are expressed as the mean ± standard error of the mean (SEM). A one-way analysis of variance followed by Dunnett’s multiple comparison test or a two-tailed *t*-test was applied using Prism 7.0 (GraphPad Software Inc., San Diego, CA, USA) to determine statistical significance. Data from the in vitro experiments are expressed as the mean ± standard deviation, analyzed using a two-tailed *t*-test. # *p* < 0.05, ## *p* < 0.01, and ### *p* < 0.001 compared with normal control or * *p* < 0.05, ** *p* < 0.01, and *** *p* < 0.001 compared with the control were considered to indicate statistical significance.

## 3. Results

### 3.1. Effects of EEVP on DNCB-Induced IgE and Histamine Levels in Serum and AD-like Skin Lesions

**Figure 1:**
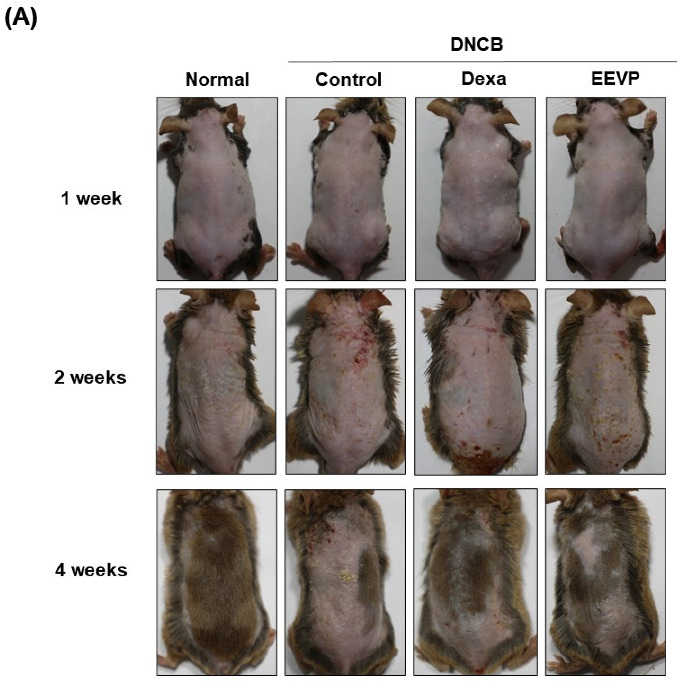

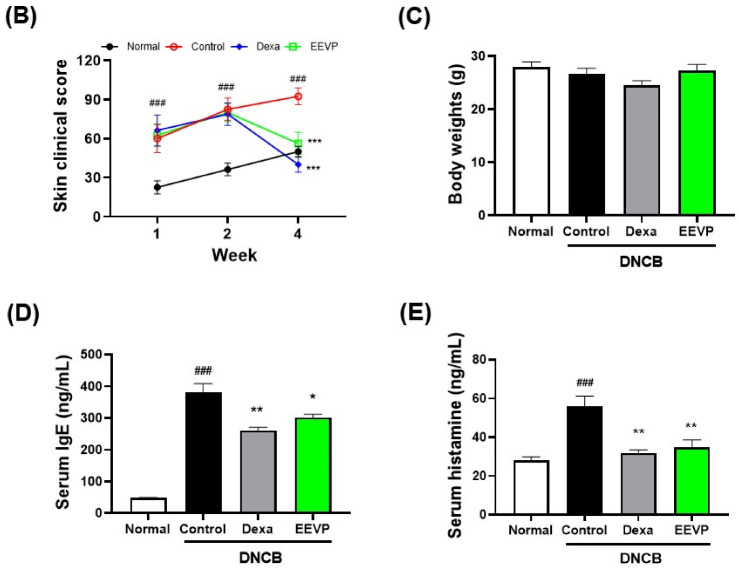
Effects of EEVP on the development of DNCB-induced AD in NC/Nga mice. (**A**) Representative image of dorsal skins of DNCB-challenged mice, (**B**) skin clinical score, (**C**) body weight, (**D**) immunoglobulin E levels, and (**E**) histamine levels in the sera of each mouse group. Normal: NC/Nga normal control; Control: DNCB-induced AD control group; Dexa: DNCB + 3 mg/kg dexame-thasone; EEVP 200: DNCB + 200 mg/kg of EEVP. Values are expressed as mean ± standard error of the mean (*n* = 6). ### *p* < 0.001 vs. Normal; * *p* < 0.05, ** *p* < 0.01, and *** *p* < 0.001 vs. Control as determined using one-way analysis of variance followed by multiple comparison tests.

We induced AD in the dorsal skins of NC/Nga mice using DNCB and investigated the pharmacological activity of EEVP against AD. Over a period of two weeks, we sensitized and challenged the mice with 0.2–1% DNCB and observed the progress of AD-like symptoms, such as erythema or eczema, on their dorsal skin. We found that DNCB caused an increase in skin clinical scores, with severe wounds, keratinization, and exfoliation on the dorsal skin in all treatment groups for up to 4 weeks, compared with those of the control group (Figure 1A,B). However, the administration of dexamethasone and EEVP significantly suppressed these skin clinical scores without any marked changes in body weight compared with that in the control group (Figure 1C). This suggests that EEVP may attenuate the severity of DNCB-induced AD-like skin allergies.

Next, we evaluated serum IgE and histamine levels to characterize EEVP activity in DNCB-induced AD. Initially, IgE levels increased in response to atopic allergens, which bind to the surface receptors of mast cells to stimulate the activity of immune cells such as B cells, monocytes, and macrophages. Histamine is a principal component of mast cell degranulation and regulates allergic inflammation at the onset of AD [35]. DNCB significantly increased serum IgE levels, and dexamethasone administration (31% decrease compared with the levels produced by DNCB) and EEVP (21% decrease compared with the levels produced by DNCB) attenuated them (Figure 1D). Similarly, serum histamine levels were significantly decreased by dexamethasone (43% decrease compared with the levels produced by DNCB) and EEVP (38% decrease compared with the levels produced by DNCB) (Figure 1E). Thus, our results suggest that the decrease in IgE and histamine levels by EEVP could reveal its inhibitory potential in reducing the inflammatory activation of AD in DNCB-induced skin inflammation.

### 3.2. Effects of EEVP on DNCB-Induced Histological Changes in Dorsal Skin

**Figure 2:**
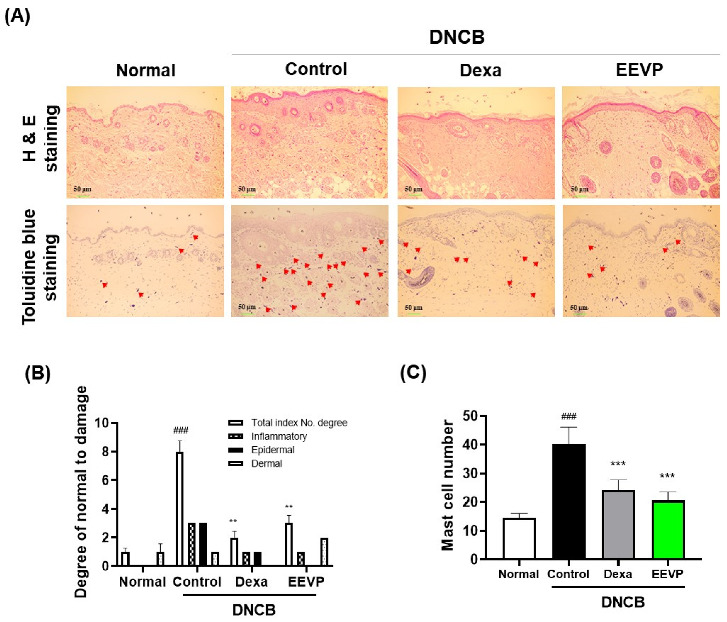
Effect of EEVP on the symptoms of AD in DNCB-challenged mice. (**A**) Hematoxylin and eosin (top) and toluidine blue (bottom) staining of dorsal skin sections. The mast cells in the dermis are indicated by red arrows. (**B**) Dermatitis score of dorsal skin section based on three clinical features (inflammatory, epidermal, or dermal). (**C**) Mast cell numbers present in a toluidine-blue-stained dorsal skin section. Normal: NC/Nga normal control; Control: DNCB-induced AD control group; Dexa: DNCB + 3 mg/kg dexamethasone; EEVP 200: DNCB + 200 mg/kg of EEVP. Values are expressed as mean ± standard error of the mean (*n* = 6). ### *p* < 0.001 vs. Normal; ** *p* < 0.01, and *** *p* < 0.001 vs. Control as determined by one-way analysis of variance followed by multiple comparison tests.

To assess the effect of EEVP on DNCB-induced skin inflammation, we conducted a histopathological analysis using H&E staining of the skin tissue (Figure 2A). We evaluated the efficacy of EEVP on AD-like symptoms, including erythema, dryness, erosion, excoriation, and hemorrhage, using dermatitis scoring of the stained skin tissue of DNCB mice. Our results revealed that the dermatitis severity score significantly increased in DNCB-induced AD-like skin but significantly decreased upon oral administration of dexamethasone or EEVP (Figure 2B).

The inhibition of IgE and serum histamine levels by EEVP suggested that EEVP may suppress the recruitment of mast cells to activate immune cells on the dorsal skin in response to DNCB. Mast cells with IgE receptors play a central role in AD by generating inflammatory factors upon IgE stimulation [36]. To confirm this, we stained the dorsal skin tissue with toluidine blue to visualize the infiltrating mast cells and counted the number of mast cells in the epidermis and dermis (Figure 2A). The repeated application of DNCB significantly increased the number of dermal mast cells. However, the administration of dexamethasone resulted in a decrease in the mast cell count of 40% compared with that produced by DNCB, and EEVP resulted in a 49% decrease, thereby markedly reducing the mast cell numbers like to those in the dexamethasone group (Figure 2C). These findings suggest that EEVP could display anti-inflammatory activity in the DNCB-induced AD model by attenuating mast cell infiltration.

### 3.3. Effects of EEVP on Immune Cell Subtypes in PBMCs, Dorsal Skin, and ALNs

**Figure 3:**
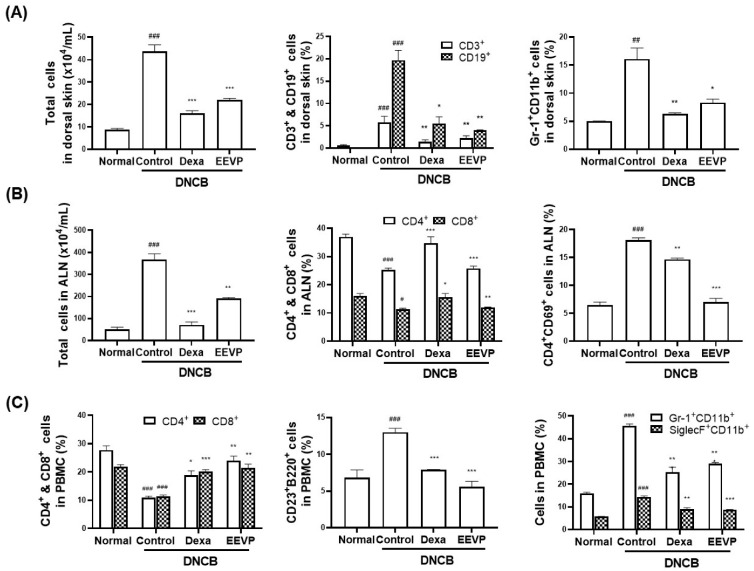
Effects of EEVP on immune cell number in NC/Nga mice with AD. (**A**) Absolute number of total cells and percentages of CD3+, CD19+, and Gr-1+CD11b+ cells in the dorsal skin. (**B**) Absolute number of total cells and percentages of CD4+, CD8+, and CD4+CD69+ cells in axillary lymph nodes. (**C**) Percentage of CD4+, CD8+, CD23+B220+, Gr-1+CD11b+, and SiglecF+CD11b+ cells in peripheral blood mononuclear cells. Normal: NC/Nga normal control; Control: DNCB-induced AD control group; Dexa: DNCB + 3 mg/kg dexamethasone; EEVP 200: DNCB + 200 mg/kg of EEVP. Values are expressed as mean ± standard error of the mean (*n* = 6). ## *p* < 0.01, and ### *p* < 0.001 vs. Normal; * *p* < 0.05, ** *p* < 0.01, and *** *p* < 0.001 vs. Control as determined using one-way analysis of variance followed by multiple comparison tests.

AD is characterized by local site inflammation accompanied by infiltration by activated inflammatory cells in the skin [37]. Given the inhibitory activity of EEVP on AD-like symptoms, such as histamine levels and infiltrated mast cell numbers, we analyzed the effect of EEVP administration on changes in the immune cell population of the dorsal skin, ALNs, and PBMCs. We showed that the total cell number increased by fourfold and the number of Gr-1+CD11b+, CD3+, and CD19+ cells increased by three- to twentyfold after DNCB challenge, compared with those of the dorsal skin in the normal group (Figure 3A). Furthermore, the total cell number and CD4+CD69+ cells increased by three- to eightfold after DNCB challenge, but CD4+ and CD8+ cell numbers were decreased by half in ALNs (Figure 3B). In PBMCs, DNCB also increased the number of CD23+B220+, Gr-1+CD11b+, and SiglecF+CD11b+ cells by twofold and decreased that of CD4+ and CD8+ cells by half (Figure 3C). However, the administration of dexamethasone and EEVP attenuated DNCB-induced changes in the total cell number and the subpopulations of immune cells in the dorsal skin, ALNs, and PBMCs.

### 3.4. Effects of EEVP on Th1 and Th2 Cytokine Levels in Splenocyte Culture Medium and Dorsal Skin

**Figure 4:**
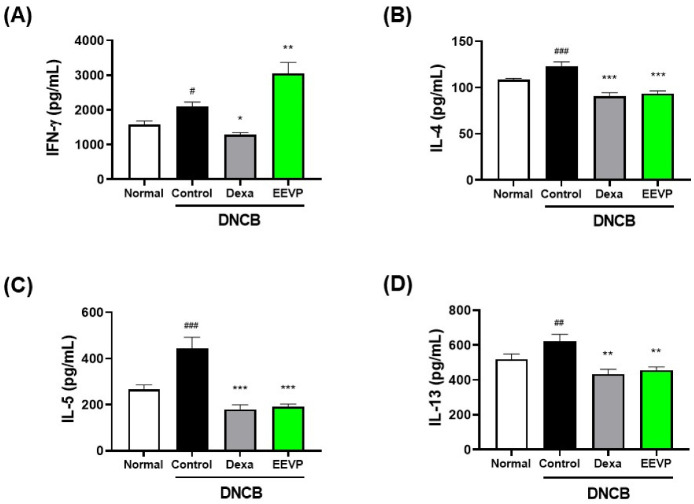
Effects of EEVP on Th1 and Th2 cytokine secretion by cultured splenocytes generated from NC/Nga mice. (**A**) Interferon (IFN)-γ, (**B**) interleukin (IL)-4, (**C**) IL-5, and (**D**) IL-13 levels were determined in culture supernatant of splenocytes incubated on anti-CD3 antibody-coating plate for 48 h with dexamethasone and EEVP. Normal: NC/Nga normal control; Control: DNCB-induced AD control group; Dexa: DNCB + 3 mg/kg dexamethasone; EEVP 200: DNCB + 200 mg/kg of EEVP. Values are expressed as mean ± standard error of the mean (*n* = 6). # *p* < 0.05, ## *p* < 0.01, and ### *p* < 0.001 vs. Normal; * *p* < 0.05, ** *p* < 0.01, and *** *p* < 0.001 vs. Control as determined using one-way analysis of variance followed by multiple comparison tests.

AD is characterized by an increased secretion of Th2 cytokines, such as IL-4, IL-5, and IL-13, whereas the generation of the Th1 mediator, including IFN-γ, is also increased in the chronic phase of AD [1,38]. Based on the regulatory activity of EEVP on the immune cell population in the dorsal skin, ALNs, and PBMCs, we examined the effect of EEVP on Th1 and Th2 cytokine production in splenocytes from DNCB mice. We found that DNCB significantly increased the secretion of Th1 (IFN-γ) and Th2 cytokines (IL-4, IL-5, and IL-13) (Figure 4). However, dexamethasone significantly decreased the secretion of IFN-γ by 39%, IL-4 by 26%, IL-5 by 59%, and IL-13 by 30% compared with that in the control. EEVP also inhibited Th2 cytokine production (IL-4 by 24%, IL-5 by 57%, and IL-13 by 27%), similarly to dexamethasone, but increased IFN-γ production by 45% compared with that of the control. These findings suggest that EEVP has a regulatory effect on the DNCB-induced imbalance in Th1/Th2 cytokine production in the spleen of the AD model.

**Figure 5:**
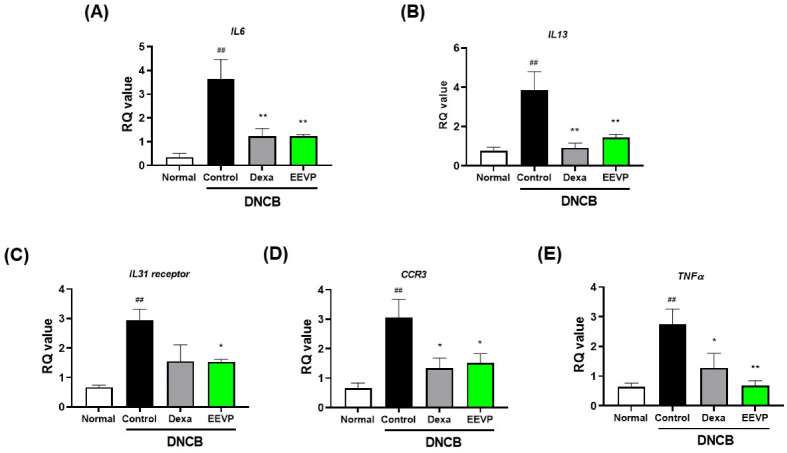
Effects of EEVP on mRNA expression levels of (**A**) *IL6*, (**B**) *IL13*, (**C**) *IL31 receptor*, (**D**) C-C motif chemokine receptor 3 (*CCR3*), and (**E**) tumor necrosis factor alpha (*TNFα*) in dorsal skin tissue of mice. Normal: NC/Nga normal control; Control: DNCB-induced AD control group; Dexa: DNCB + 3 mg/kg dexamethasone; EEVP 200: DNCB + 200 mg/kg of EEVP. Values are expressed as mean ± standard error of the mean (*n* = 6). ## *p* < 0.01 vs. Normal; * *p* < 0.05, and ** *p* < 0.01 vs. Control as determined by one-way analysis of variance followed by multiple comparison tests.

DNCB exposure in the skin induces the infiltration of immune cells, including activated T cells, to produce cytokines, chemokines, and chemokine receptors associated with the development of AD. Thus, we investigated the potential effect of EEVP on regulating the mRNA expression levels of inflammatory factors in skin. We found that DNCB significantly increased the mRNA expression levels of Th2 cytokines (*IL6* and *IL13*), cytokine receptors (*IL31* receptor), chemokine receptors (C-C motif chemokine receptor 3 (*CCR3*), and Th1 cytokine (*TNFα*) (Figure 5). However, EEVP significantly decreased the mRNA levels of these genes by one- to threefold, similarly to dexamethasone.

### 3.5. Effects of EEVP on Expression of Inflammatory Genes and the Nrf2/HO-1 Pathway in HaCaT Keratinocytes

**Figure 6:**
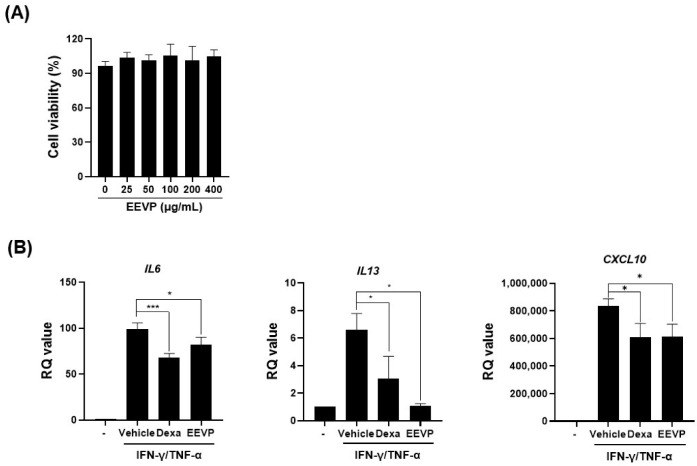
Effects of EEVP on mRNA expression of IFN-γ/TNF-α-stimulated HaCaT cells. (**A**) Cell viability and (**B**) mRNA expression levels of *IL6*, *IL13*, and C-X-C motif chemokine ligand-10 (*CXCL10*) were analyzed using quantitative real-time polymerase chain reaction. Data are expressed as mean ± standard deviation (*n* = 3). Vehicle: DMSO-treated cells; Dexa: dexamethasone (20 μM)-treated cells; EEVP: EEVP (400 μg/mL)-treated cells. * *p* < 0.05, and *** *p* < 0.001 as determined using two-tailed *t*-tests.

When exposed to allergens, skin keratinocytes are the principal cells that secrete inflammatory cytokines and chemokines that activate skin-resident immune cells. In vitro stimulation of HaCaT keratinocytes with IFN-γ/TNF-α induces the expression of inflammatory cytokines such as IL-4, IL-6, and IL-13, resulting in immune cell infiltration into the epidermis, resembling AD pathogenesis [39]. Therefore, we examined the effect of EEVP on IFN-γ/TNF-α-induced mRNA expression of inflammatory factors in HaCaT keratinocytes. We found that IFN-γ/TNF-α stimulation significantly induced the mRNA expression of *IL6*, *IL13*, and C-X-C motif chemokine ligand 10 (*CXCL10*). However, dexamethasone and EEVP significantly suppressed the mRNA expression of *IL6*, *IL13*, and *CXCL10* without affecting cell viability (Figure 6). This result was similar to that of EEVP on the dorsal skin (Figure 5A,B), which indicate the reliability of HaCaT cells as an AD-like in vitro model. Notably, there was a discrepancy between the inhibitory effect of EEVP on the mRNA expression of *IL6* (14% less of a decrease) and *IL13* (30% more of a decrease) compared with that in the dexamethasone group. This may reflect the limitation of the herbal extract when compared directly with that of the pharmacological molecule.

**Figure 7:**
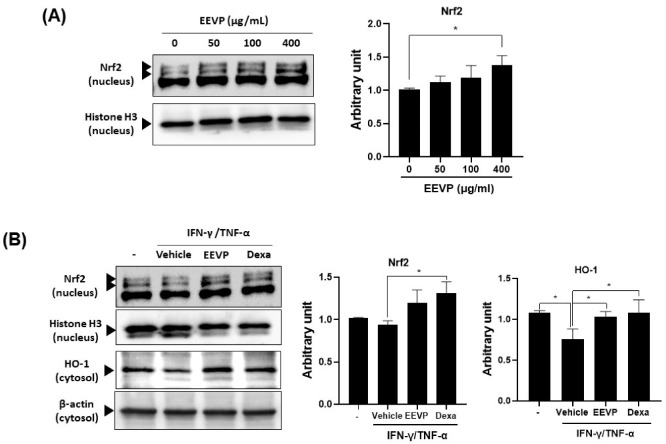
Stimulatory effects of EEVP on Nrf2 and HO-1 expression in HaCaT cells. (**A**) Nrf2 expression in the nucleus was examined in HaCaT cells after EEVP (0, 50, 100, and 400 μg/mL) treatments. (**B**) Nrf2 and HO-1 expression in the nucleus and cytosol, respectively, were examined in HaCaT cells after EEVP (400 μg/mL) treatment. Vehicle: DMSO-treated cells; Dexa: dexamethasone (20 μM)-treated cells; EEVP: EEVP-treated cells. Quantified Western blot data are shown as the mean ± standard deviation (*n* = 3). * *p* < 0.05 as determined using two-tailed *t*-tests.

Oxidative stress damages the epithelial cells in the skin and induces an inflammatory response in AD-like skin diseases. Nrf2 is a master regulator of the expression of detoxifying enzymes and antioxidant proteins, such as HO-1, in response to oxidative stress on keratinocytes [14]. The Nrf2/HO-1 pathway limits skin inflammation by inhibiting the generation of inflammation cytokines [40,41]. Therefore, we investigated the possible molecular pathway underlying EEVP inhibition in AD via the regulation of Nrf2 expression in HaCaT keratinocytes. We found that EEVP increased Nrf2 expression in HaCaT keratinocytes in a dose-dependent manner (Figure 7A). Next, we explored whether the expression of HO-1 was affected by EEVP in IFN-γ/TNF-α-stimulated HaCaT keratinocytes. Notably, EEVP induced Nrf2 expression, although not as significantly as dexamethasone, but significantly restored HO-1 expression attenuated by IFN-γ/TNF-α treatment, similarly to that shown by dexamethasone (Figure 7B). Additionally, we found that EEVP increased HO-1 expression in the dorsal skin of DNCB-treated mice.

### 3.6. Docking Analysis of Five Components in EEVP

**Figure 8:**
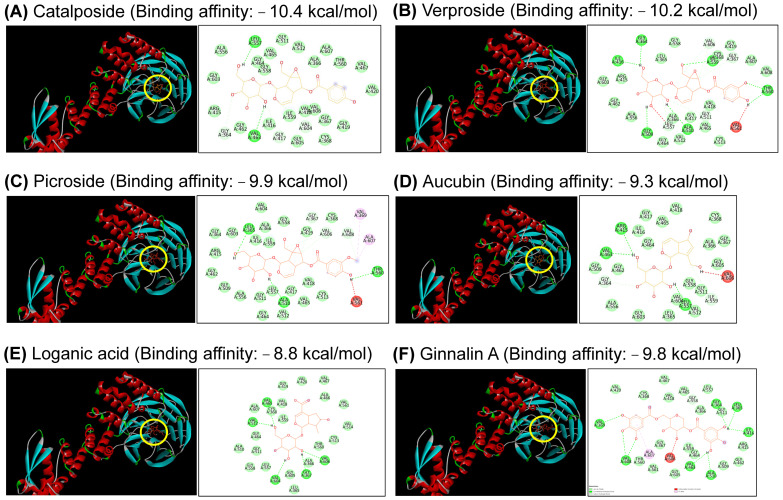
Molecular docking results between the structure of the Keap1 Kelch domain pocket and five components of EEVP: (**A**) catalposide, (**B**) verproside, (**C**) picroside, (**D**) aucubin, (**E**) loganic acid, and (**F**) ginnalin A. The binding position of each component in the docking pocket of the Kelch domain is shown by a yellow circle.

Nrf2 dissociation from the Kelch domain of the Keap1 by binding ginnalin A results in the nuclear translocation of Nrf2, which increases Nrf2-regulated antioxidant molecules, such as HO-1 protein [42]. Thus, we analyzed the molecular docking of five components (catalposide, verproside, picroside II, aucubin, and loganic acid) in EEVP and ginnalin A (as a positive control) with the Kelch domain pocket of Keap1, using the molecular docking prediction software. We found that the binding affinity scores of catalposide, verproside, and picroside II were −10.4, −10.2, and −9.9, respectively (Figure 8A–C), which were higher than that of ginnalin A (−9.8 kcal/mol) (Figure 8F). The binding affinity scores of aucubin and loganic acid were −8.8 and −6.9, respectively, which were sufficient for binding with the Kelch domain, although lower than that of ginnalin A (Figure 8D,E).

## 4. Discussion

An immune imbalance in the Th1/Th2 system reflects a specific feature of AD pathogenesis. In AD, IL-4 is essential for Th2 cell development and IgE production via Ig class switching in B cells [43,44]. The IgE level is a direct parameter for assessing the degree or progression of disease severity in AD-like inflammatory diseases [36]. The significance of IgE is supported by the ability of IgE-selective immunoadsorption therapy to improve the condition of patients with severe AD during the treatment period [45]. In addition, IL-5 participates in the maturation and infiltration of eosinophils associated with allergic reactions [46]. Early in a response to tissue injury, IL-6 induces pro-inflammatory cytokine release from tissue-resident immune cells such as keratinocytes [47], but IL-13 is a key cytokine that drives inflammation in the periphery at the tissue level [48]. IL-31 and its receptor are associated with itching and scratching behavior in AD mice [49]. IFN-γ induces the hyper-responsive release of cytokines in the keratinocytes of patients in the chronic phase of AD [38]. Thymic stromal lymphopoietin (TSLP) from keratinocytes interacts with Th2 cells to produce IL-4 in patients with AD [50]. TSLP also binds to TRPA1-positive sensory neurons, triggering itching in AD mice [51]. We found that EEVP inhibited DNCB-induced TSLP expression in dorsal skin. Therefore, EEVP activity regulates the Th1/Th2 balance in the spleen as a primary immune organ, which exerts an effect on inflammatory mediators in peripheral tissues such as the skin. This suggests that EEVP inhibitory activity on inflammatory factors might contribute to alleviating the skin inflammatory response observed in the DNCB-induced AD model.

The infiltration by immune cells such as T cells, B cells, and eosinophils is observed in the skin of patients with AD [37,52]. In addition, lymph nodes adjacent to the affected skin become enlarged owing to the increased influx of immune cells [53], and various immune cell types have an activated phenotype in PBMCs. The increased numbers of activated T and B cells involved in adaptive immune responses, as well as dendritic cells and macrophages that participate in antigen presentation, have also been suggested to enhance the immune response in the ALNs of a mouse model [54,55]. We found that EEVP lowered the number of various inflammatory cells including T cells (CD3+, CD4+, CD8+, CD4+/CD69+), IgE-producing CD23+B220+ B cells, and eosinophils (Gr-1+CD11b+) in dorsal skin, ALNs, and PBMCs of an AD mouse model, similar to that in a previous report [25]. Thus, our results suggest that EEVP attenuates the infiltration of activated inflammatory cells into the ALNs and skin in response to DNCB-induced allergic responses in AD.

Oxidative stress in the skin induces an inflammatory response in AD-like skin diseases. The Nrf2/HO-1 pathway plays a key role in the regulation of skin inflammation by inhibiting the generation of inflammation cytokines [15,40]. HO-1 plays a protective role against experimental skin wounds, psoriasis, and inflammatory AD [18,56]. The function of HO-1 in the skin has been attributed to its ability to attenuate antigen presentation by dendritic cells and the production of chemokines and cytokines by keratinocytes [57,58]. We found that EEVP attenuated the pro-inflammatory response in AD-like dorsal skin and partially induced Nrf2 expression, but significantly induced HO-1 expression under IFN-γ/TNF-α-treatment conditions. Recently, mice with enhanced Nrf2 activation were found to exhibit a disruption in the epidermis barrier and hyperkeratosis, resulting in an ichthyosis-like skin disease phenotype, suggesting that limited Nrf2 activation is beneficial for the skin under stressful conditions [59]. Our findings suggest that EEVP exerts anti-inflammatory and protective effects on skin cells by attenuating pro-inflammatory cytokine and chemokine production by keratinocytes, partially via the Nrf2/HO-1 signaling pathway. Therefore, EEVP may improve AD-like dermal lesions by inducing the Nrf2/HO-1 pathway to attenuate oxidative stress and suppress inflammation, thereby restoring skin homeostasis.

Skin barrier function is properly regulated for skin homeostasis upon pathogen or environmental stress. Aryl hydrocarbon receptor (AhR) is a ligand-dependent transcription factor necessary for the regulation of skin barrier-related proteins such as filaggrin in keratinocytes [60,61]. Medicinal coal tar and glyteer act as anti-oxidative AhR agonists to activate AhR and Nrf2, which increases anti-oxidative enzyme levels and neutralizes IL-13/IL-4-mediated ROS generation [62,63]. Furthermore, tapinarof activates both AhR and Nrf2, upregulates filaggrin and involucrin expression, and exhibits anti-oxidative activity [64]. Therefore, to elucidate the potential activity of EEVP in the skin barrier-related signaling pathway, the effect of EEVP on the AhR pathway should be explored in a future study.

The clinical signs of AD frequently precede the development of asthma and allergic rhinitis, known as the allergic triad. TSLP, an epithelial cell-derived cytokine, has been suggested as a candidate to influence the Th2 response in lung- and skin-specific allergic disorders [50,65]. We found that EEVP inhibits the DNCB-induced TSLP level in the skin of mice. EEVP also has an inhibitory effect on house dust mite-induced asthmatic inflammation by regulating Th1/Th2 inflammatory factors [22]. Therefore, the potential effect of EEVP on TSLP and the relevant mechanism linking the allergic triad might be investigated in the future.

The strong binding affinity of catalposide and verproside to the Kelch domain of Keap1 contributes to the induction of HO-1 expression by *Veronica ciliata* Fisch. in BRL-3A hepatocyte cells [66]. Additionally, catalposide protects hydrogen peroxide-induced neuronal cell death by inducing HO-1 expression [67]. Picroside II also enhances HO-1 activity in testicular oxidative stress induced by an ischemia/reperfusion rat model [68]. Thus, these findings suggest that EEVP components may bind the pocket of the Kelch domain of Keap1, thereby upregulating the Nrf2/HO-1 pathway. The results of the docking analysis provide evidence that EEVP is an effective treatment for AD. The docking-based in silico experiment was the process of finding the drug action point of the Nrf2/HO-1 signaling pathway. Subsequently, in vitro experiments showed that Nrf2 and HO-1 expression were increased at the cellular level. In addition, in vivo experiments showed the effectivity of EEVP for AD. The significance of this study is that it identified the pharmacological mechanism of EEVP acting on AD via in silico, in vitro, and in vivo experiments and demonstrated its potential as an AD drug. Further studies are needed to identify the unknown active molecules involved and their reciprocal action mechanisms.

## 5. Conclusions

This study is the first to provide evidence that EEVP mitigates DNCB-induced AD inflammation in a murine model by attenuating immune cell activation and inflammatory cytokine production, thereby restoring immune homeostasis. Furthermore, our findings indicate that the immune regulatory pathway of EEVP may mediate the induction of Nrf2/HO-1 signaling, which might attenuate oxidative stress in the skin. These results demonstrate the potential of EEVP as an effective and novel pharmacological medicine for patients with AD.

## Data Availability

The data presented in this study are available in the article.
